# Malignant recto-jejunal fistula: a rare case report

**DOI:** 10.1097/MS9.0000000000002301

**Published:** 2024-06-21

**Authors:** Roshna Adhikari, Mukesh Paudel, Ram M. Jha, Raman K. Sah, Saroj Sitaula, Prabhat Silwal

**Affiliations:** a Department of Radiology, National Academy of Medical Sciences, Bir Hospital; b Department of Anesthesiology, National Academy of Medical Sciences, Bir Hospital, Kathmandu; c Department of Internal Medicine, BP Koirala Institute of Health Sciences, Dharan, Nepal

**Keywords:** case report, digestive system fistula, malignancy, rectal carcinoma

## Abstract

**Introduction and importance:**

Malignancy can lead to colo-enteric fistulas. A malignant fistula between the rectum and the jejunum is a rare occurrence.

**Case presentation:**

A 60-year-old female suffered from diarrhea, vomiting, and epigastric pain for 4 months. After demonstration of a dilated rectum with heterogeneous collection on ultrasonography, contrast-enhanced computed tomography (CECT) along with rectal contrast was done, which showed heterogeneously enhancing asymmetrical circumferential thickening of the proximal rectum, including rectosigmoid junction, collection in the rectum and two recto-jejunal fistulous tracts. Colonoscopy showed ulcero-proliferative growth in the rectum with two fistulous tracts communicating with the jejunum. Biopsy from the growth indicated a poorly differentiated adenocarcinoma. Conservative and palliative treatment was provided.

**Clinical discussion:**

Clinical features of colo-enteric fistulas can include abdominal pain, diarrhea, and weight loss. The patient may be asymptomatic in some cases. Options for diagnosis include barium studies, enteroscopy, colonoscopy, CECT, and computed tomography enterography (CTE). Malignant bowel fistula is associated with serious complications resulting in high morbidity and mortality rates. Surgical resection and fistula repair are the mainstay of curative treatment.

**Conclusion:**

Long-standing gastrointestinal symptoms like chronic diarrhea in the elderly should be investigated with imaging modalities like CECT. Early detection with imaging can reduce debilitating metabolic and nutritional deficiencies and improve patient’s morbidity and mortality.

## Introduction

Highlights

- Recto-jejunal fistula secondary to malignancy is a rare occurrence.
- Presenting symptoms can be subtle and may include symptoms like diarrhea and abdominal pain.
- Long-standing gastrointestinal symptoms in the elderly should be thoroughly investigated to rule out gastrointestinal malignancy.
- Early detection of malignancy with imaging can reduce debilitating metabolic derangements and nutritional deficiencies and improve patient’s morbidity and mortality.

Acquired gastrointestinal fistulas can be classified as internal, external, and mixed^1^. In a patient with an internal gastro-intestinal fistula, communication exists between segments of the intestine or with any other hollow viscus, whereas in the case of an external gastro-intestinal fistula, the intestine communicates with the skin of the abdominal wall^1^. The principal causes of spontaneous internal enteroenteric fistulas in order of frequency are Crohn’s Disease, diverticular disease, colorectal malignancy, radiation enteritis, tuberculosis, and actinomycosis^1–3^.

Among colo-enteric fistulas, a colo-duodenal fistula between the proximal transverse colon and duodenum is common due to the close proximity of these organs^4^. Recto-jejunal and colo-jejunal fistulas are rarer. A thorough search on the PubMed database revealed only a few cases of malignant recto-jejunal and colo-jejunal fistulas^2,4–7^.

Malignant rectojejunal fistula is a very rare occurrence with unknown exact incidence^5^. Route of spread is postulated as direct invasion of jejunum by colorectal cancer^5^. Several cases of benign coloenteric fistulas have been reported, with very few reported cases of malignant recto jejunal and colo-jejunal fistula^2,4–6^.

Here, we present a case of a 60-year-old female with a malignant recto-jejunal fistula. We discuss the interplay of clinical evaluation, radiological imaging, and histopathological examination in the diagnosis. This case report has been prepared in accordance with the case report (CARE) guidelines^8^.

## Case presentation

A 60-year-old female presented to the emergency department with symptoms of intermittent diarrhea, vomiting, and epigastric pain for 4 months. She did not suffer from any chronic illness, did not smoke, and did not consume alcohol. Her general physical examination, vital signs, and systemic examination all revealed normal findings.

The patient was initially managed with antiemetics, intravenous fluid resuscitation, close observation, and regular monitoring of vital signs. Blood tests revealed a white cell count of 12.7×109/l, hemoglobin was 11.4 gm/dl, and platelet count was 335 000/cumm. Serum urea and creatinine, electrolytes, liver function tests, and microscopic examination of stool were all within the normal range.

**Figure 1:**
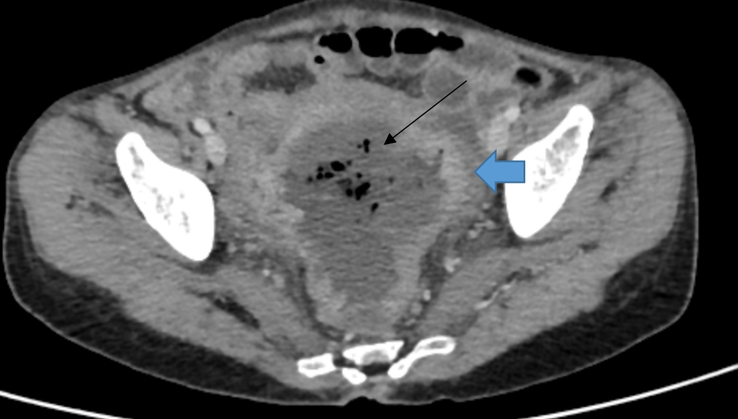
Axial section of contrast-enhanced computed tomography (CECT) of abdomen and pelvis showing heterogeneously enhancing asymmetrical circumferential thickening of the proximal rectum and rectosigmoid junction (blue bold arrow) and dilated rectum with heterogeneous collection (black thin arrow).

**Figure 2:**
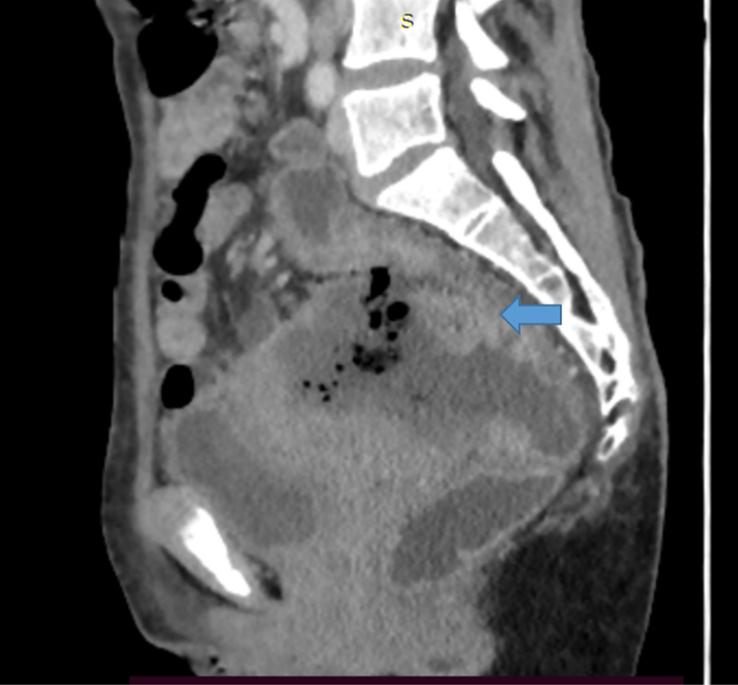
Sagittal section of contrast-enhanced computed tomography (CECT) of abdomen and pelvis showing heterogeneously enhancing asymmetrical circumferential thickening of the proximal rectum and rectosigmoid junction (blue bold arrow).

**Figure 3:**
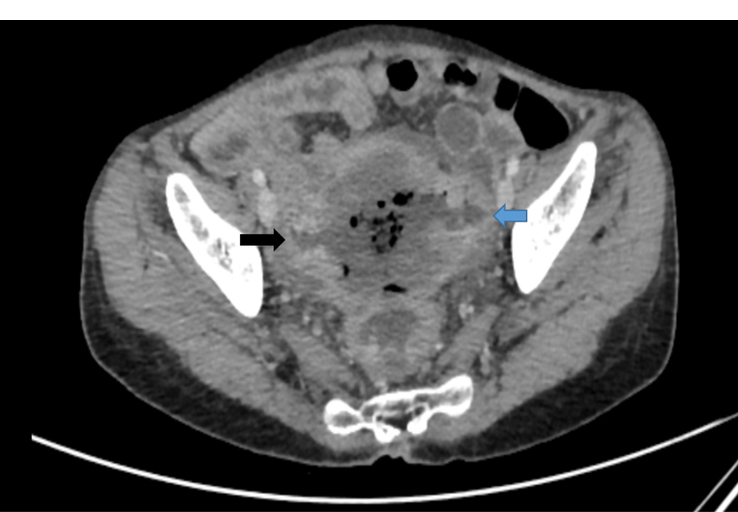
Axial section of contrast-enhanced computed tomography (CECT) showing two recto-jejunal fistulas, on the right (black arrow) and left (blue arrow) side.

**Figure 4:**
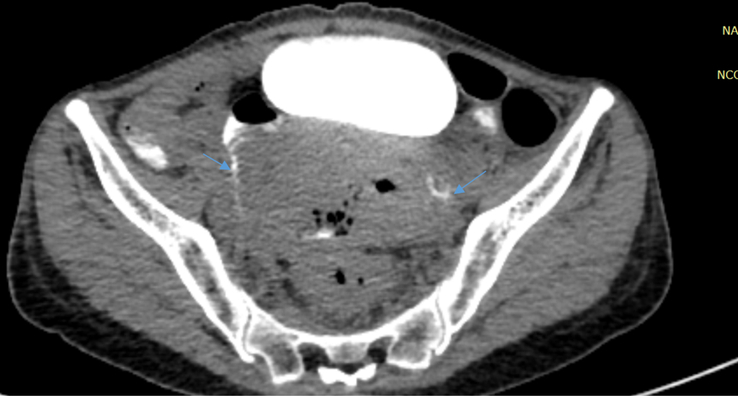
Axial section of computed tomography (CT) of abdomen and pelvis with rectal contrast showing thin rim of contrast passing from rectum to jejunal loops on bilateral sides via the fistulas (thin blue arrows).

**Figure 5:**
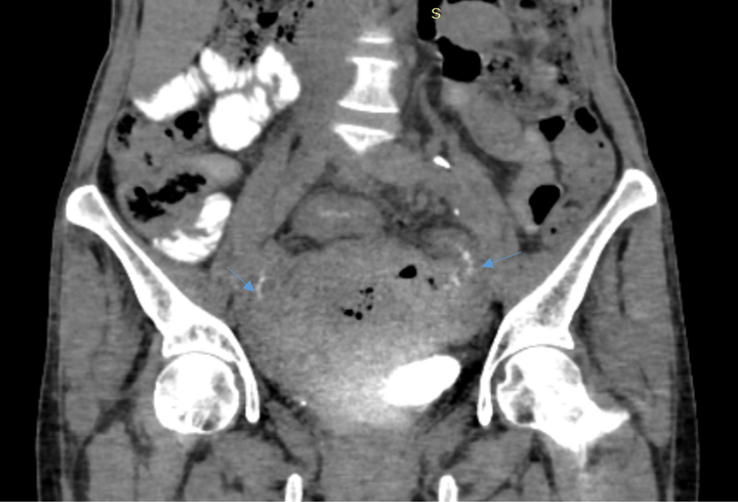
Coronal section of computed tomography (CT) of abdomen and pelvis with rectal contrast showing a thin rim of contrast passing from the rectum to jejunal loops on bilateral sides via the fistulas (thin blue arrows).

**Figure 6:**
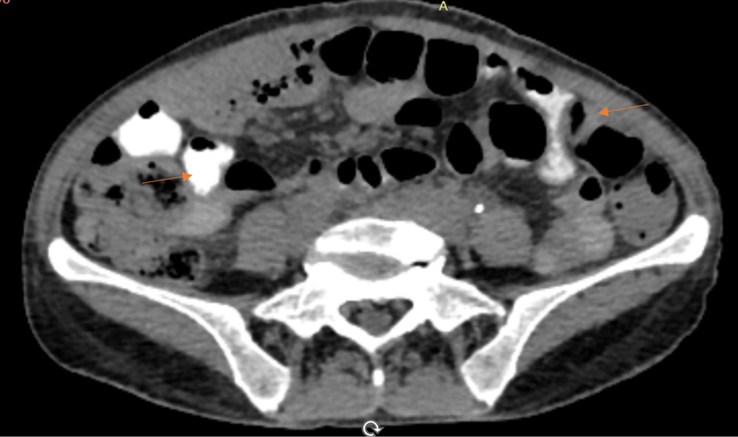
Axial section of contrast-enhanced computed tomography (CECT) showing filling of jejunal loops with contrast via the recto-jejunal fistulas (Orange thin arrows).

**Figure 7:**
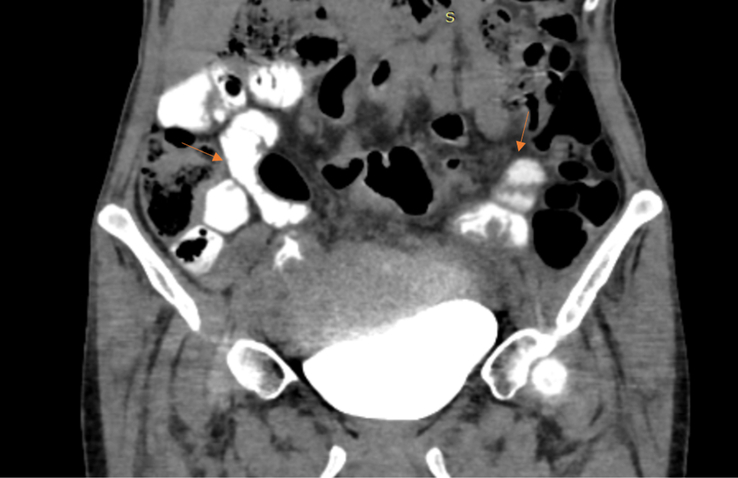
Coronal section of contrast-enhanced computed tomography (CECT) showing filling of jejunal loops with contrast via the recto-jejunal fistulas (Orange thin arrows).

Plain abdominal and chest films were unremarkable, with no free air under the diaphragm or bowel dilatation. Ultrasonography of the abdomen and pelvis revealed a dilated rectum with a heterogeneous collection (with multiple air foci within). Following this, contrast-enhanced computed tomography of the abdomen and pelvis (CECT) was done along with rectal contrast. CECT showed heterogeneously enhancing asymmetrical circumferential thickening of the proximal rectum, including the rectosigmoid junction with surrounding areas of fat strandings. The rectum was distended with a heterogeneous collection containing multiple air foci as shown in Figure 1 and Figure 2. Two fistulous tracts were noted between the jejunum and the rectum, measuring 5 mm and 7 mm. The contrast was noted to travel from the rectum to the jejunum in the rectal contrast study, as depicted in Figure 3, Figure 4, and Figure 5. Hence, filling of jejunal loops with contrast via the recto-jejunal fistulas was noted, as shown in Figure 6 and Figure 7.

**Figure 8:**
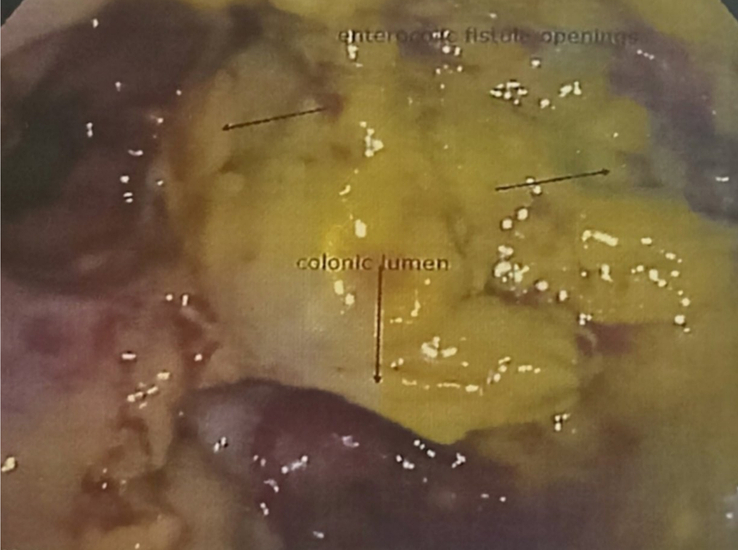
Photograph taken during colonoscopy showing two separate openings from the colon in continuation with small bowel lumen.

Following this, the patient underwent a flexible full-length colonoscopy and biopsy. Colonoscopy revealed an ulcero-proliferative growth ~15–20 cm proximal to the anal verge. Two separate openings were seen in continuation with the small bowel lumen as seen in Figure 8. A biopsy was obtained from the growth.

**Figure 9:**
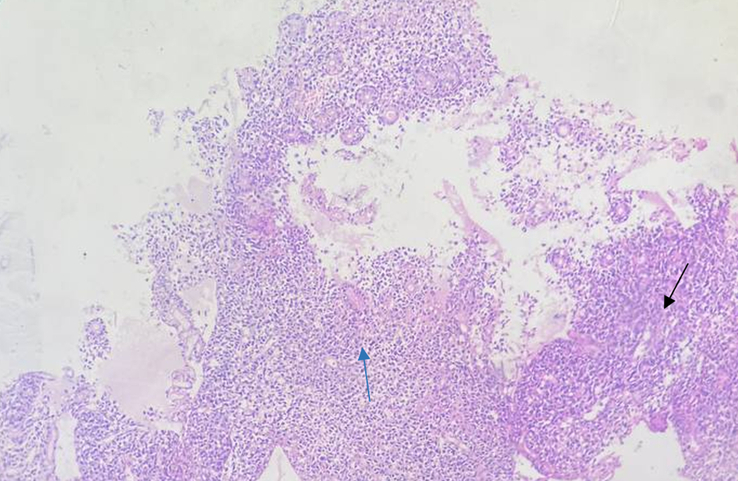
Photograph taken during histopathological examination of rectal biopsy, showing poorly. differentiated adenocarcinoma (Black thin arrow showing growth/mass and blue thin arrow showing mitosis).

Histopathological sections showed multiple fragments of monotonous tumor cells arranged in poorly formed glands and sheets infiltrating the stroma. Individual cells showed moderate pleomorphism with irregular nuclei and moderate amount of cytoplasm. The nucleo-cytoplasmic ratio was increased. Ulceration, necrotic inflammatory debris, and chronic inflammatory infiltrates were also seen within fragments of colonic mucosa. All these findings as seen in Figure 9 were in favor of a diagnosis of poorly differentiated adenocarcinoma.

Patient and the patient’s family members were counseled about the nature of the disease and the poor prognosis. Treatment options including surgical and conservative options, the possible outcomes and the associated risks were all explained. The patient and the family opted for conservative management. The patient’s symptoms stabilized during her hospital stay. She was discharged on her 10th day of admission. She was prescribed an oral rehydration solution, tablet antiemetics, and a proton pump inhibitor. After discharge, the patient gradually deteriorated. The patient received palliative care at her home and passed away 25 days later.

## Discussion

Few cases of colo-jejunal fistula have been reported previously. Noronha *et al*.^5^, reported a case of malignant colo-jejunal fistula with the primary lesion being a carcinoma of the distal transverse colon and proximal descending colon. Torosian *et al*.^4^, reported a case of malignant colo-jejunal fistula, with the primary being carcinoma of the distal transverse colon. Mahajan *et al*.^2^, reported a case of malignant colo-jejunal fistula with the primary being adenocarcinoma of the rectosigmoid junction. Chun *et al*.^6^, reported another rare case of malignant colo-jejunal fistula secondary to lymphoma of the distal transverse colon.

Clinical manifestation of gut-to-gut fistula can be subtle. Abdominal pain, diarrhea, weight loss, and fever are the common symptoms^3,9^. Cases of such fistulas may also be clinically silent and detected accidentally on imaging^5^. Our patient had initially presented with abdominal pain, diarrhea, and vomiting.

The degree of diarrhea associated with this type of fistula is often severe. Several hypotheses have been postulated to account for the etiology of this diarrhea. These include (1) regurgitation of colonic flora into the small intestine with subsequent malabsorption secondary to bacterial enteritis, (2) rapid shunting of intraluminal contents into the colon, and (3) colonic irritation secondary to unconjugated bile acid^4^.

Options for diagnosis include barium studies, enteroscopy, colonoscopy, CECT, and computed tomography enterography (CTE)^10^. Barium studies, enteroscopy, and colonoscopy have limitations as they are mainly luminal studies. CECT is more accurate and demonstrates both luminal and extraluminal pathology like abscesses, bowel wall inflammation, and metastases. CTE, which uses a negative oral contrast medium, has higher sensitivity in detecting luminal pathology and subtle mucosal lesions than routine CECT, which uses a positive oral contrast medium. However, growth and small abscesses may be missed in CTE^5,10^.

Malignant bowel fistula is associated with serious complications resulting in high morbidity and mortality rates. Advanced malignancy itself and fistula-related complications like fluid and electrolyte imbalances, malnutrition, abscess, sepsis, multiorgan failure, and bleeding reduce the patient’s survival^5^.

The primary treatment of malignant colo-enteric fistula is surgical^4,11^. However, it is noteworthy that the morbidity and mortality is high despite surgical resection and the risk of complications during the surgery is high^3,12^. Operative surgical resection of underlying obstructive malignancy can also be performed as a form of palliative treatment^5,12^. Our patient and her family opted for conservative and palliative management.

The choice of conservative over definitive treatment in our case is a limitation of our case study. Nevertheless, we believe this study is valuable as it highlights the pattern of presentation, the interplay of various investigations for diagnosis, and therapeutic challenges in cases of malignant recto-jejunal fistula.

## Conclusion

Malignant colo-enteric fistula is a rare entity and among that recto-jejunal fistula is extremely rare. Clinical presentation is subtle with detection primarily on imaging. CTE is superior to CECT for detection of the fistula, whereas CECT is superior for extraluminal pathologies like malignant growth and abscess. Long-standing gastrointestinal symptoms like chronic diarrhea in the elderly should be investigated with imaging modalities like CECT. Early detection with imaging can reduce debilitating metabolic and nutritional deficiencies and improve patient’s morbidity and mortality. Similar case reports in the future with different modalities of treatment and different outcomes may help us better understand the best treatment modalities for patients.

## Ethical approval

At our institution, case reports require written informed consent from the patient which has been obtained. However, case reports do not require ethical review from the institutional review committee at our institution.

## Consent

Written informed consent was obtained from the patient for publication of this case report and accompanying images. A copy of the written consent is available for review by the Editor-in-Chief of this journal on request.

## Source of funding

This work did not receive any funding. There are no sources of funding to be declared.

## Author contribution

R.A., M.P., and R.M.J.: contributed to patient care, conceptualization, design, writing the original draft, and reviewing and editing; R.A., M.P., R.M.J., R.K.S., S.S., and P.S.: contributed to contributed to design, writing the original draft, and reviewing and editing. All the authors have approved the final version for publication.

## Conflicts of interest disclosure

The authors declare that they have no financial or personal conflicts of interest with regard to the content of this publication.

## Research registration unique identifying number (UIN)

Not applicable.

## Guarantor

Roshna Adhikari.

## Data availability statement

All data that support the findings of this study are included in this article. Further inquiries can be directed to the corresponding author.
